# Global trends of antimicrobial susceptibility to ceftaroline and ceftazidime–avibactam: a surveillance study from the ATLAS program (2012–2016)

**DOI:** 10.1186/s13756-020-00829-z

**Published:** 2020-10-27

**Authors:** Hui Zhang, Yingchun Xu, Peiyao Jia, Ying Zhu, Ge Zhang, Jingjia Zhang, Simeng Duan, Wei Kang, Tong Wang, Ran Jing, Jingwei Cheng, Yali Liu, Qiwen Yang

**Affiliations:** Department of Clinical Laboratory, Peking Union Medical College Hospital, Peking Union Medical College, Chinese Academy of Medical Science, Beijing, 100730 China

**Keywords:** Ceftaroline, Ceftazidime–avibactam, Antibiotics, Surveillance, Antimicrobial resistance

## Abstract

**Background:**

This study reports the global trends of antimicrobial susceptibility to ceftaroline and ceftazidime–avibactam using data from the Antimicrobial Testing Leadership and Surveillance (ATLAS) program between 2012 and 2016.

**Methods:**

For the 2012–2016 ATLAS program, 205 medical centers located in Africa-Middle East (n = 12), Asia–Pacific (n = 32), Europe (n = 94), Latin America (n = 26), North America (n = 31), and Oceania (n = 10) consecutively collected the clinical isolates. The minimum inhibitory concentrations (MICs) and in vitro susceptibilities to ceftaroline and ceftazidime–avibactam were assessed using the Clinical and Laboratory Standards Institute (CLSI) 2019and European Committee on Antimicrobial Susceptibility Testing (EUCAST) 2019 guidelines.

**Results:**

Between 2012 and 2016, 176,345 isolates were collected from around the globe and included in the analysis. Regarding Gram-negative bacteria, ceftazidime–avibactam demonstrated high susceptibility (> 90%) against *Enterobacteriaceae* and *Pseudomonas aeruginosa*, with increased antimicrobial activity observed from the addition of avibactam (4 mg/L) to ceftazidime. Regarding Gram-positive bacteria, ceftaroline showed > 90% susceptibility against *Staphylococcus aureus*, *Streptococcus pneumoniae*, α-and β-hemolytic *Streptococcus*. The antimicrobial susceptibilities to ceftaroline and ceftazidime–avibactam were mostly stable from 2012 to 2016, but the susceptibilities to ceftazidime–avibactam to carbapenem-resistant (CR) *Klebsiella pneumonia* (88.4–81.6%) and to CR-*P. aeruginosa* (89.6–72.7%) decreased over time. In terms of regional difference, the susceptibilities of methicillin-resistant *S. aureus* to ceftaroline in Asia and of CR-*K. pneumonia* to ceftazidime–avibactam in Asia/Africa-Middle East were lower compared with other regions, while the susceptibility of CR-*P. aeruginosa* to ceftazidime–avibactam in North America was higher.

**Conclusion:**

The addition of avibactam improves the activity of ceftazidime against *Enterobacteriaceae* and *P. aeruginosa*. The global antimicrobial susceptibilities to ceftaroline and ceftazidime–avibactam were, in general, stable from 2012 to 2016, but a marked reduction in the susceptibilities of specific species and CR-*P. aeruginosa* to ceftazidime–avibactam was observed.

## Introduction

The rapidly increasing and global spreading of the resistance of bacteria to antibiotics in recent years is a serious challenge for clinicians and a global health crisis [1]. Multi-drug resistance in both Gram-negative and -positive bacteria often leads to untreatable infections using conventional antibiotics, and even last-resort antibiotics are losing their power [2]. The increases in the occurrence of infections caused by third-generation cephalosporin- and carbapenem-resistant (CR)-*Enterobacteriaceae*, CR-*Pseudomonas aeruginosa*, and CR-*Acinetobacter baumannii* are of particular concern since they are associated with tremendously increased mortality and morbidityrates [3, 4]. Recently, the World Health Organization has rated CR-*Enterobacteriaceae*, CR-*P. aeruginosa*, and CR-*A. baumannii* as top critical-priority resistant bacteria, outweighing methicillin-resistant *Staphylococcus aureus* [5]. Consequently, updated epidemiological data on antibiotic resistance is needed to adapt the treatment strategies to the reality, which changes at an alarming rate [4, 6–8].

Ceftaroline is a fifth-generation broad-spectrum cephalosporin. It is mainly active against methicillin-resistant *S. aureus* and Gram-positive bacteria, but also against Gram-negative bacteria [9]. Ceftarolineis indicated for community-acquired pneumonia and complicated skin infections [10-13]. Avibactam is a diazabicyclooctane derivative antibiotic that can reversibly inhibit β-lactamase enzymes, including Ambler class A (ESBL and KPC), class C, and partial class D (including OXA-1, OXA-10, and OXA-48-like) enzymes by covalent acylation of the active-site serine residue [14]. Ceftazidime–avibactam is a novel β-lactam/β-lactamase inhibitor combination that has shown potency against a wide variety of CR-*Enterobacteriaceae*. Ceftazidime–avibactam has been approved for the management of complicated urinary tract infections, complicated intra-abdominal infections, hospital-acquired pneumonia, and infections from aerobic Gram-negative bacteria with limited treatment options [15].

Ceftaroline and ceftazidime–avibactam are relatively novel antibiotics that show promises in the control of antibiotic-resistant pathogens. They are readily available around the globe. The patterns of resistance to ceftaroline and ceftazidime–avibactam around the globe remain to be defined exactly and represent crucial data for monitoring global health threats. Therefore, this study aimed to: (1) examine the in vitro activities of ceftaroline, ceftazidime–avibactam, and various comparative agents from 2012 to 2016 using the data from a global antibiotic surveillance program, the Antimicrobial Testing Leadership And Surveillance (ATLAS) program; and (2) compare the susceptibility profile of various pathogen species over time and across different regions of the world, with an emphasis on antibiotic-resistant pathogens.

## Materials and methods

### Bacterial isolates

For the 2012–2016 ATLAS program, 205 medical centers located in Africa-Middle East (n = 12), Asia–Pacific (n = 32), Europe (n = 94), Latin America (n = 26), North America (n = 31), and Oceania (n = 10) contributed to the consecutive collection of clinical isolates. The specimens were obtained from inpatients with specific types of infections (skin and skin structure infection, intra-abdominal infection, urinary tract infection, lower respiratory tract infection, and blood infection). The pathogens were isolated and identified by each participating center, stored in tryptic soy broth with glycerol at − 70 °C, and shipped to International Health Management Associates, Inc. (IHMA; Schaumburg, IL, USA) for susceptibility testing. The present study only included the isolates considered to be the potential pathogen of the patient’s infection. If multiple samples were taken from the same patient during an infectious event, only the first positive sample for this infectious event was included in the ATLAS program. The pathogen identification was confirmed by MALDI-TOF at IHMA (Schaumburg, IL, USA) prior to susceptibility testing. Methicillin-resistant *S. aureus* is defined in this study as *S. aureus* resistant to oxacillin.

### Antimicrobial susceptibility testing

IHMA (Schaumburg, IL, USA) carried out all antimicrobial susceptibility tests using the broth microdilution method. The minimum inhibitory concentrations (MICs) were interpreted using the Clinical and Laboratory Standards Institute (CLSI) 2019 and the European Committee on Antimicrobial Susceptibility Testing (EUCAST) 2019 breakpoints [16, 17]. Tigecycline was interpreted using the Food and Drug Administration and EUCAST 2019 interpretative breakpoints. Ceftaroline, ceftazidime–avibactam (avibactam at a fixed concentration of 4 mg/L), and the following comparator agents were tested:ceftazidime, cefepime, penicillin, ampicillin, piperacillin–tazobactam, doripenem, imipenem, meropenem, levofloxacin, moxifloxacin, clindamycin, erythromycin, vancomycin, teicoplanin, linezolid, daptomycin, gentamicin, tigecycline, minocycline, trimethoprim–sulfamethoxazole, amikacin, colistin, aztreonam, quinupristin–dalfopristin, andoxacillin. In the present study, the data were analyzed for *Escherichia coli*, *Klebsiella pneumoniae*, *Enterobactercloacae*, *Citrobacter freundii*, *Proteus mirabilis*, *P. aeruginosa*, *A. baumannii*,* S. aureus*, *Streptococcus pneumoniae*, α- and β-hemolytic *Streptococcus*, coagulase-negative *Staphylococcus*, *Enterococcus faecalis*, and *Enterococcus faecium*, as well as resistant species including CR-*E. coli*, CR-*K. pneumoniae*, *CR-Enterobacter cloacae*, CR-*P. aeruginosa*, CR-*A. baumannii*, methicillin-resistant *S. aureus*, and penicillin-resistant *S. pneumoniae*. All tests included quality control strains from the American Type Culture Collection (ATCC; Manassas, VA, USA). *Escherichia coli* ATCC 25922, *K. pneumoniae* ATCC 700603, *P. aeruginosa* ATCC 27853, *S. aureus* ATCC 29213, and *S. pneumoniae* ATCC 49619 were used for quality control according to the CLSI 2019 guidelines. All quality control results were within the published ranges.

## Results

### Sample retrieval

A total of 176,345 isolates were collected between 2012 and 2016. The numbers of isolates of each species group tested are listed in Tables 1 and 2. The largest number of isolates were collected from patients > 60 years (82,518, 46.8%) and 31–60 years (59,428, 33.7%), followed by patients < 18 years (19,446, 11.0%) and 19–30 years (13,350, 7.6%). Regarding the infection types, 64,032 (36.3%) isolates were collected from skin and skin structure infections, 52,077 (29.5%) from lower respiratory tract infections, 26,868 (15.2%) from urinary tract infections, 12,847 (7.3%) from intra-abdominal infections, and 11,930 (6.8%) from the blood. In regard to hospital location, 74,554 (42.3%), 32,430 (18.4%), 17,024 (9.7%), 16,339(9.3%), 10,130(5.7%), and 8200 (4.6%) isolates were from patients in the general medical wards, general surgical wards, emergency rooms, medical intensive care unit (ICUs), surgical ICUs, and general pediatric wards, respectively.

**Table 1:** In vitro susceptibilities of Gram-negative strains obtained from the ATLAS program, 2012–2016

Organism/antibiotic	No. of isolates	MIC_50_	MIC_90_	MIC range	CLSI^a^			EUCAST		
					S%	I%	R%	S%	I%	R%
*Escherichia coli*										
Ceftaroline	21903	0.12	256	0.015–256	66.5	2.6	30.9	66.5	0	33.5
Ceftazidime–avibactam	21903	0.12	0.25	0.015–256	99.8	0	0.2	99.8	0	0.2
Ceftazidime	21903	0.25	32	0.015–256	79.2	3.0	17.8	74.0	5.1	20.8
Cefepime	21903	0.12	32	0.12–32	76.2	4.7	19.1	74.6	3.6	21.8
Pip-taz	21903	2	16	0.25–256	90.3	4.6	5.1	84.8	5.4	9.8
Doripenem	21903	0.03	0.06	0.008–16	99.6	0.1	0.3	99.6	0.1	0.3
Imipenem	21903	0.25	0.25	0.03–16	99.1	0.4	0.5	99.5	0.4	0.2
Meropenem	21903	0.03	0.06	0.004–16	99.5	0.1	0.4	99.6	0.2	0.2
Levofloxacin	21903	0.25	16	0.004–16	62.3	1.7	36.0	58.8	2.8	38.4
Tigecycline	21903	0.25	0.5	0.015–16	99.8	0.2	0	99.0	0.8	0.2
Amikacin	21903	2	8	0.25–64	98.2	0.9	0.9	94.5	3.7	1.8
Colistin	13964	0.5	1	0.06–16	NA	NA	NA	99.5	0	0.5
Aztreonam	21903	0.12	64	0.015–256	76.0	3.1	20.9	72.3	3.7	24.0
*Klebsiella pneumoniae*										
Ceftaroline	18114	0.25	256	0.015–256	57.5	2.0	40.5	57.5	0	42.5
Ceftazidime–avibactam	18114	0.12	1	0.015–256	98.8	0	1.2	98.8	0	1.2
Ceftazidime	18114	0.25	128	0.015–256	64.3	1.9	33.8	61.6	2.7	35.7
Cefepime	18114	0.12	32	0.12–32	65.1	6.0	28.9	63.7	3.2	33.1
Pip/taz	18114	4	256	0.25–256	73.0	7.8	19.2	64.4	8.6	27.0
Doripenem	18114	0.06	0.5	0.008–16	91.6	1.0	7.4	91.6	1.0	7.4
Imipenem	18114	0.25	1	0.03–16	90.3	1.9	7.8	92.2	2.4	5.5
Meropenem	18114	0.06	0.5	0.004–16	91.1	1.1	7.9	92.1	2.0	5.9
Levofloxacin	18114	0.12	8	0.004–16	73.2	3.1	23.7	61.8	9.1	29.1
Tigecycline	18114	0.5	2	0.015–16	96.4	3.1	0.5	88.2	8.2	3.6
Amikacin	18114	1	8	0.25–64	93.6	3.0	3.4	91.0	2.6	6.4
Colistin	12884	0.5	1	0.06–16	NA	NA	NA	96.3	0	3.7
Aztreonam	18114	0.12	256	0.015–256	64.2	1.0	34.8	62.4	1.8	35.8
*Enterobacter cloacae*										
Ceftaroline	4330	0.5	256	0.015–256	60.0	3.1	37.0	60.0	0.0	40.1
Ceftazidime–avibactam	4330	0.25	1	0.015–256	97.8	0.0	2.2	97.8	0.0	2.2
Ceftazidime	4330	0.5	128	0.015–256	67.3	1.4	31.3	64.0	3.3	32.7
Cefepime	4330	0.12	32	0.12–32	78.5	8.8	12.7	73.3	10.8	15.9
Pip-taz	4330	4	256	0.25–256	75.3	8.0	16.7	69.9	5.4	24.7
Doripenem	4330	0.06	0.25	0.008–16	96.8	0.4	2.9	96.8	0.4	2.9
Imipenem	4330	0.5	1	0.03–16	93.1	3.5	3.4	96.6	2.0	1.5
Meropenem	4330	0.06	0.12	0.004–16	96.8	0.6	2.6	97.4	1.3	1.3
Levofloxacin	4330	0.06	4	0.004–16	88.8	2.8	8.4	80.8	5.7	13.5
Tigecycline	4330	0.5	1	0.015–16	96.3	3.2	0.5	90.1	6.2	3.7
Amikacin	4330	2	4	0.25–64	97.6	0.9	1.6	96.0	1.6	2.4
Colistin	2889	0.5	1	0.12–16	NA	NA	NA	93.7	0.0	6.3
Aztreonam	4330	0.12	64	0.015–256	68.2	1.4	30.4	65.8	2.4	31.8
*Citrobacter freundii*										
Ceftaroline	2327	0.25	128	0.015–256	61.9	2.1	36.0	61.9	0	38.1
Ceftazidime–avibactam	2327	0.12	0.5	0.015–256	98.5	0	1.5	98.5	0	1.5
Ceftazidime	2327	0.5	128	0.015–256	68.0	1.9	30.1	64.3	3.8	32.0
Cefepime	2327	0.12	4	0.12–32	89.8	3.6	6.7	84.4	7.3	8.4
Pip-taz	2327	4	128	0.25–256	77.1	12.0	11.0	70.5	6.6	23.0
Doripenem	2327	0.06	0.12	0.008–16	97.9	0.3	1.8	97.9	0.3	1.8
Imipenem	2327	0.5	2	0.03–16	88.9	8.5	2.6	97.4	2.1	0.5
Meropenem	2327	0.03	0.06	0.004–16	97.7	0.5	1.8	98.2	1.2	0.6
Levofloxacin	2327	0.12	4	0.008–16	87.0	4.0	9.0	76.5	6.2	17.3
Tigecycline	2327	0.5	1	0.015–8	98.9	1.1	0	94.9	4.0	1.1
Amikacin	2327	2	4	0.25–64	98.4	0.4	1.2	97.1	1.3	1.6
Colistin	1593	0.5	1	0.06–16	NA	NA	NA	99.6	0	0.4
Aztreonam	2327	0.25	64	0.015–256	69.2	2.4	28.4	66.2	3.1	30.8
*Proteus mirabilis*										
Ceftaroline	3950	0.12	128	0.015–256	79.4	2.0	18.6	79.4	0	20.6
Ceftazidime–avibactam	3950	0.03	0.06	0.015–256	99.7	0	0.3	99.7	0	0.3
Ceftazidime	3950	0.06	1	0.015–256	95.2	1.7	3.1	91.1	4.1	4.8
Cefepime	3950	0.12	8	0.12–32	88.2	3.4	8.5	86.9	2.9	10.3
Pip-taz	3950	0.5	1	0.25–256	98.5	0.9	0.6	97.7	0.8	1.5
Doripenem	3950	0.25	0.5	0.008–16	98.4	1.0	0.6	98.4	1.0	0.6
Imipenem	3950	2	4	0.03–16	25.8	45.9	28.3	71.7	27.7	0.6
Meropenem	3950	0.06	0.12	0.004–16	99.6	0.2	0.3	99.8	0.2	0.1
Levofloxacin	3950	0.12	8	0.015–16	76.6	5.5	17.9	64.9	4.5	30.6
Tigecycline	3950	2	8	0.03–16	52.2	37.3	10.5	20.9	31.3	47.8
Amikacin	3950	4	8	0.25–64	95.6	1.1	3.4	91.5	4.1	4.4
Colistin	2412	16	16	0.25–16	NA	NA	NA	0.5	0	99.5
Aztreonam	3950	0.015	0.5	0.015–256	95.9	0.8	3.3	93.1	2.9	4.1
*Pseudomonas aeruginosa*										
Ceftaroline	16014	16	256	0.015–256	NA	NA	NA	NA	NA	NA
Ceftazidime–avibactam	16014	2	8	0.015–256	91.9	0	8.1	91.9	0	8.1
Ceftazidime	16014	4	64	0.06–256	76.7	4.6	18.8	76.7	0	23.4
Cefepime	16014	4	32	0.12–32	78.4	11.2	10.5	78.4	0	21.6
Pip-taz	16014	8	256	0.25–256	68.9	13.8	17.3	68.9	0	31.2
Doripenem	16013	0.5	8	0.008–16	74.3	7.6	18.2	67	7.2	25.8
Imipenem	16014	2	16	0.03–16	63.4	8.2	28.4	71.6	4.5	23.9
Meropenem	16014	0.5	16	0.008–16	72.5	6.0	21.5	72.5	11.9	15.6
Levofloxacin	16014	1	8	0.004–16	70.4	6.8	22.9	61.7	0	38.3
Amikacin	16014	4	16	0.25–64	90.4	2.7	6.9	85.9	4.5	9.6
Colistin	12449	1	2	0.06–16	96.6	0	3.4	96.6	0	3.4
Aztreonam	16014	8	32	0.015–256	NA	NA	NA	3.9	73.4	22.8
*Acinetobacter baumannii*										
Ceftaroline	3567	256	256	0.015–256	NA	NA	NA	NA	NA	NA
Ceftazidime–avibactam	3567	32	128	0.03–256	NA	NA	NA	NA	NA	NA
Ceftazidime	3567	64	256	0.015–256	30.1	2.4	67.5	NA	NA	NA
Cefepime	3567	32	32	0.12–32	29.9	10.4	59.7	NA	NA	NA
Pip-taz	3567	256	256	0.25–256	25.4	3.7	70.9	NA	NA	NA
Doripenem	3567	8	16	0.015–16	33.2	1.4	65.4	30.4	2.8	66.8
Imipenem	3567	16	16	0.03–16	33.8	1.2	65.0	33.8	2.7	63.5
Meropenem	3567	16	16	0.015–16	32.8	1.6	65.6	32.8	3.5	63.7
Levofloxacin	3567	8	16	0.03–16	29	9.6	61.4	26.1	1.0	73
Tigecycline	3567	1	2	0.015–16	NA	NA	NA	NA	NA	NA
Amikacin	3567	64	64	0.25–64	42.5	5.8	51.7	40.2	2.3	57.5
Colistin	2404	1	2	0.06–16	94.3	0	5.7	94.3	0	5.7
Aztreonam	3567	64	256	0.015–256	NA	NA	NA	NA	NA	NA

*CLSI* Clinical Laboratory and Standards Institute, *EUCAST* European Committee on Antimicrobial Susceptibility Testing, *NA* not applicable

^a^Cefepime CLSI susceptibility for *Enterobacteriaceae *adopted the susceptible, susceptible-dose-dependent, and resistant categories

**Table 2:** In vitro susceptibilities of Gram-positive strains obtained from the ATLAS program, 2012–2016.

Organism/antibiotic	No. of isolates	MIC_50_	MIC_90_	MIC range	CLSI			EUCAST		
					S%	I%	R%	S%	I%	R%
*Staphylococcus aureus*										
Ceftaroline	50525	0.5	1	0.015–64	93.4	6.2	0.4	93.4	6.2	0.4
Ceftazidime–avibactam	50525	32	64	0.015–64	NA	NA	NA	NA	NA	NA
Ceftazidime	50525	32	64	0.015–64	NA	NA	NA	NA	NA	NA
Pip-taz	50525	8	32	0.12–32	NA	NA	NA	NA	NA	NA
Levofloxacin	50525	0.5	8	0.015–8	56.9	0.4	42.7	56.9	0	43.1
Moxifloxacin	50525	0.12	4	0.008–8	57.1	2.7	40.1	56.8	0	43.2
Tigecycline	50525	0.12	0.25	0.015–4	98.9	1.1	0	98.9	0	1.1
Minocycline	50525	0.12	1	0.12–16	93.2	3.4	3.4	89.4	1.4	9.2
Gentamicin	31019	0.5	64	0.06–64	85	0.7	14.3	56.1	0	43.9
Daptomycin	50525	0.5	1	0.06–4	99.8	0.2	0	99.8	0	0.2
Trimethoprim sulfa	31019	0.25	1	0.25–8	96.8	0	3.3	96.8	0.7	2.6
Teicoplanin	50525	0.5	1	0.12–32	100	0	0	98.1	0	1.9
Vancomycin	50525	1	2	0.25–4	100	0	0	100	0	0
Clindamycin	50525	0.12	4	0.03–8	74.8	0.3	24.9	74.2	0.6	25.2
Erythromycin	50525	1	16	0.12–16	48	3.4	48.6	50.6	0.3	49.1
Linezolid	50525	2	2	0.5–16	100	0	0	100	0	0
Oxacillin	50525	4	8	0.06–8	40.4	0	59.6	NA	NA	NA
*Streptococcus pneumoniae*										
Ceftaroline	11005	0.008	0.12	0.004–32	99.7	0.3	0	98.7	0	1.3
Ceftazidime–avibactam	11005	0.25	16	0.015–128	NA	NA	NA	NA	NA	NA
Ceftazidime	11005	0.25	16	0.015–128	NA	NA	NA	NA	NA	NA
Doripenem	11005	0.015	1	0.015–8	98	2	0	98	0	2
Meropenem	11005	0.015	1	0.008–2	78	9.2	12.8	100	0	0
Levofloxacin	11005	1	1	0.12–16	98.5	0.2	1.3	98.5	0	1.5
Moxifloxacin	11005	0.12	0.25	0.03–8	98.5	0.5	1.1	98.4	0	1.6
Tigecycline	11005	0.03	0.03	0.008–2	99.9	0.1	0	NA	NA	NA
Minocycline	11005	0.12	4	0.015–4	71.3	5.1	23.6	69.6	1.7	28.7
Daptomycin	11005	0.25	0.5	0.03–8	NA	NA	NA	NA	NA	NA
Vancomycin	11005	0.25	0.5	0.008–2	100	0	0	100	0	0
Clindamycin	11005	0.06	2	0.008–2	74.8	0.4	24.8	75.2	0	24.8
Erythromycin	11005	0.06	2	0.008–2	64.3	0.3	35.4	64.3	0.3	35.4
Linezolid	11005	1	2	0.06–4	100	0	0	100	0	0
Penicillin	11005	0.03	2	0.015–16	61.8	20.7	17.5	61.8	28.9	9.3
*α-hemolytic Streptococcus*										
Ceftaroline	12138	0.008	0.12	0.004–32	99.7	0.3	0	98.7	0	1.3
Ceftazidime–avibactam	12138	0.25	16	0.015–128	NA	NA	NA	NA	NA	NA
Ceftazidime	12138	0.25	16	0.015–128	NA	NA	NA	NA	NA	NA
Penicillin	12138	0.03	2	0.015–16	NA	NA	NA	NA	NA	NA
Doripenem	12138	0.015	1	0.015–8	NA	NA	NA	98	0	2
Meropenem	12138	0.015	1	0.008–2	NA	NA	NA	100	0	0
Levofloxacin	12138	1	2	0.12–16	98.3	0.3	1.4	98.5	0	1.5
Moxifloxacin	12138	0.12	0.25	0.03–8	98.5	0.5	1.1	98.4	0	1.6
Minocycline	12138	0.12	4	0.015–4	NA	NA	NA	69.6	1.7	28.7
Tigecycline	12138	0.03	0.03	0.008–2	NA	NA	NA	100	0	0
Clindamycin	12138	0.06	2	0.008–2	75.6	0.4	24.1	75.9	0	24.1
Erythromycin	12138	0.06	2	0.008–2	64.8	0.3	34.9	64.3	0.3	35.4
Vancomycin	12138	0.5	0.5	0.008–2	100	0	0	100	0	0
Linezolid	12138	1	2	0.06–8	100	0	0	100	0	0
Daptomycin	12138	0.25	0.5	0.03–8	99.3	0.7	0	100	0	0
*β-hemolytic Streptococcus*										
Ceftaroline	9019	0.004	0.015	0.004–1	100	0	0	NA	NA	NA
Ceftazidime–avibactam	9019	0.12	0.5	0.015–128	NA	NA	NA	NA	NA	NA
Ceftazidime	9019	0.12	0.5	0.015–128	NA	NA	NA	NA	NA	NA
Penicillin	9019	0.015	0.06	0.015–8	NA	NA	NA	NA	NA	NA
Doripenem	9019	0.015	0.03	0.015–8	NA	NA	NA	100	0	0
Meropenem	9019	0.015	0.06	0.008–2	99.9	0.1	0	100	0	0
Levofloxacin	9019	0.5	1	0.12–16	98.3	0.2	1.5	98.2	0	1.9
Moxifloxacin	9019	0.12	0.25	0.03–8	NA	NA	NA	98.1	0	1.9
Minocycline	9019	0.12	4	0.015–4	69.9	30.1	0	65.6	0.9	33.4
Tigecycline	9019	0.03	0.06	0.008–2	100	0	0	100	0	0
Clindamycin	9019	0.06	0.12	0.008–2	90.6	0.3	9	91	0	9
Erythromycin	9019	0.06	2	0.008–2	83.4	0.7	15.8	84.4	0.6	15
Vancomycin	9019	0.5	0.5	0.008–1	100	0	0	100	0	0
Linezolid	9019	1	2	0.06–8	100	0	0	100	0	0
Daptomycin	9019	0.12	0.5	0.03–8	100	0	0	100	0	0
*CoNS*										
Ceftaroline	8490	0.25	1	0.015–64	NA	NA	NA	NA	NA	NA
Ceftazidime–avibactam	8490	16	64	0.015–64	NA	NA	NA	NA	NA	NA
Ceftazidime	8490	16	64	0.015–64	NA	NA	NA	NA	NA	NA
Pip-taz	8490	2	32	0.12–32	NA	NA	NA	NA	NA	NA
Levofloxacin	8490	4	8	0.015–8	46.9	1.8	51.4	46.9	0	53.1
Moxifloxacin	8490	1	4	0.008–8	NA	NA	NA	NA	NA	NA
Minocycline	8490	0.25	0.5	0.12–16	48.9	14.6	36.5	46.5	0	53.5
Tigecycline	8490	0.25	0.5	0.015–4	98.7	1.3	0	98.7	0	1.3
Clindamycin	8490	0.12	8	0.03–8	65.5	2.1	32.4	63.7	1.9	34.5
Erythromycin	8490	8	16	0.12–16	33.1	1.1	65.8	33.4	0.3	66.3
Vancomycin	8490	1	2	0.25–8	99.9	0.1	0	99.9	0	0.1
Teicoplanin	8490	2	8	0.12–64	98	1.7	0.3	85	0	15
Linezolid	8490	1	2	0.5–16	99.4	0	0.6	99.4	0	0.6
Daptomycin	8490	0.5	1	0.06–4	99.6	0.4	0	99.6	0	0.4
Gentamicin	5336	2	64	0.06–64	54.7	5.5	39.8	33.8	0	66.3
Trimethoprim sulfa	5336	1	8	0.25–8	61	0	39	61	10	28.9
Oxacillin	8490	4	8	0.06–8	25.7	0	74.3	NA	NA	NA
*Enterococcus faecalis*										
Ceftaroline	3194	1	16	0.015–64	NA	NA	NA	NA	NA	NA
Ceftazidime–avibactam	3194	64	64	1–64	NA	NA	NA	NA	NA	NA
Ceftazidime	3194	64	64	1–64	NA	NA	NA	NA	NA	NA
Levofloxacin	3194	1	16	0.06–16	68	1.1	30.8	NA	NA	NA
Tigecycline	3194	0.12	0.25	0.015–4	94.1	5.9	0	94.1	3.9	2
Minocycline	3194	16	16	0.06–16	25.8	13.7	60.5	NA	NA	NA
Daptomycin	3194	2	4	0.06–8	99.8	0.2	0	NA	NA	NA
Teicoplanin	3194	0.5	0.5	0.12–64	98.3	0.1	1.7	97.8	0	2.2
Vancomycin	3194	1	2	0.12–64	94.3	3.8	1.9	94.3	0	5.7
Erythromycin	3194	16	16	0.06–16	14.6	27.4	58	NA	NA	NA
Linezolid	3194	1	2	0.06–8	99.3	0.6	0.2	99.8	0	0.2
Quinupristin dalfopristin	2014	8	16	0.25–16	1	7.7	91.3	NA	NA	NA
*Enterococcus faecium*										
Ceftaroline	2546	64	64	0.03–64	NA	NA	NA	NA	NA	NA
Ceftazidime–avibactam	2546	64	64	0.12–64	NA	NA	NA	NA	NA	NA
Ceftazidime	2546	64	64	0.12–64	NA	NA	NA	NA	NA	NA
Levofloxacin	2546	16	16	0.06–16	12.2	3.9	83.9	NA	NA	NA
Tigecycline	2546	0.12	0.25	0.015–8	95.5	4.5	0	95.5	3	1.5
Minocycline	2546	2	16	0.06–16	55.4	12.4	32.2	NA	NA	NA
Daptomycin	2546	4	4	0.06–16	98	2	0	NA	NA	NA
Teicoplanin	2546	1	64	0.12–64	76	1	23	75.2	0	24.8
Vancomycin	2546	1	64	0.12–64	69.2	5	25.8	69.2	0	30.8
Erythromycin	2546	16	16	0.06–16	3.6	11.2	85.2	NA	NA	NA
Linezolid	2546	1	2	0.06–16	97.4	2.5	0.2	99.8	0	0.2
Quinupristin dalfopristin	1577	1	4	0.06–16	73.2	13.9	12.9	NA	NA	NA

*CLSI* Clinical Laboratory and Standards Institute, *EUCAST* European Committee on Antimicrobial Susceptibility Testing, *NA* not applicable, *CoNS* coagulase-negative staphylococci

### In vitro activities of ceftaroline and ceftazidime–avibactam against Gram-negative bacteria from 2012 to 2016

Table 1 (Gram-negative) and 2 (Gram-positive) show the in vitro activities of ceftaroline, ceftazidime–avibactam, and comparators against the selected bacteria. Ceftazidime–avibactam demonstrated high activities against all tested Gram-negative bacteria (CLSI/EUCAST 2019 susceptibility, 91.9–99.8%). The susceptibility of *A. baumannii* was not calculated because of the absence of a breakpoint, but the MICs of this antibiotic were higher for *A. baumannii* than for the other bacteria (MIC_50_/MIC_90,_ 32/128 mg/L). The addition of avibactam drastically increased the activity of ceftazidime against *E. coli*, *K. pneumoniae*, *E. cloacae*, *C. freundii*, and *P. aeruginosa* (CLSI 2019 susceptibilities to ceftazidime alone, 64.3–79.2%) whereas a trend of decreased MIC was observed for *A. baumannii*, as indicated by a twofold reduction in MIC_90_ (ceftazidime, MIC_50_/MIC_90,_ 64/256 mg/L). Regarding the comparator agents, the susceptibility of *Enterobacteriaceae* was, in general, high for carbapenems and tigecycline (> 90%). For *A. baumannii*, the most potent antibiotics were colistin and tigecycline (MIC_50_/MIC_90_, 1/2 mg/L), with aMIC_50_ of ≥ 8 and aMIC_90_ of ≥ 16 mg/L observed for all other tested agents.

Regarding resistant Gram-negative strains, the activities of ceftazidime–avibactam were moderate for CR-*E. coli* (MIC_50_/MIC_90_, 0.5/256 mg/L), CR-*K. pneumoniae* (MIC_50_/MIC_90_, 1/256 mg/L), and CR-*P. aeruginosa* (MIC_50_/MIC_90_, 4/64 mg/L) and low for *CR-E. cloacae* and CR-*A. baumannii* (MIC_50_/MIC_90,_ 64–128/256 mg/L) (Table 3). Regarding the comparator agents, the susceptibilities of CR-*E. coli*, CR-*K. pneumoniae*, *CR-E. cloacae*, CR-*P. aeruginosa*, and CR-*A. baumannii* were low for the vast majority of the tested antibiotics. Good potency was observed for tigecycline against all tested *Enterobacteriaceae* (MIC_50_/MIC_90,_ 0.25–1/1–4 mg/L), and for colistin against CR-*E. coli*, *CR-E. cloacae*, CR-*P. aeruginosa*, and CR-*A. baumannii* (MIC_50_/MIC_90,_ 0.5–1/1–2 mg/L).

**Table 3:** In vitro susceptibilities of multi-drug resistant strains obtained from the ATLAS program, 2012–2016.

Organism/antibiotic	No. of isolates	MIC_50_	MIC_90_	MIC range	CLSI^a^			EUCAST		
					S%	I%	R%	S%	I%	R%
*CRECO*										
Ceftaroline	119	256	256	0.015–256	10.9	1.7	87.4	9.5	0	90.5
Ceftazidime–avibactam	119	0.5	256	0.03–256	72.3	0	27.7	40.5	0	59.5
Ceftazidime	119	64	256	0.12–256	22.7	4.2	73.1	11.9	2.4	85.7
Cefepime	119	32	32	0.12–32	8.4	16	75.6	0	2.4	97.6
Pip-taz	119	256	256	0.5–256	21.9	5.9	72.3	19.1	2.4	78.6
Doripenem	119	4	16	0.03–16	32.8	11.8	55.5	19.1	2.4	78.6
Imipenem	119	8	16	4–16	0	0	100	0	0	100
Meropenem	119	8	16	0.015–16	26.1	6.7	67.2	19.1	7.1	73.8
Levofloxacin	119	8	16	0.015–16	24.4	3.4	72.3	7.1	2.4	90.5
Tigecycline	119	0.25	1	0.03–4	98.3	1.7	0	88.1	7.1	4.8
Amikacin	119	8	64	1–64	78.2	5	16.8	59.5	7.1	33.3
Colistin	79	0.5	1	0.12–16	NA	NA	NA	96.3	0	3.7
Aztreonam	119	64	256	0.015–256	23.5	0.8	75.6	16.7	2.4	81
*CRKPN*										
Ceftaroline	1418	256	256	0.06–256	0.6	0	99.4	0	0	100
Ceftazidime–avibactam	1418	1	256	0.015–256	85.6	0	14.4	83.6	0	16.4
Ceftazidime	1418	256	256	0.12–256	4	2.5	93.5	0.6	0.7	98.7
Cefepime	1418	32	32	0.12–32	3.5	8	88.6	0.4	0.6	99
Pip-taz	1418	256	256	2–256	1.5	1.1	97.4	0.3	0.1	99.6
Doripenem	1418	8	16	0.03–16	4.2	5.4	90.4	0.7	0.4	98.9
Imipenem	1418	16	16	4–16	0	0	100	0	0	100
Meropenem	1418	16	16	0.015–16	2.9	4	93.1	0.8	3.9	95.3
Levofloxacin	1418	8	16	0.03–16	12.7	3.5	83.9	3.1	1.6	95.2
Tigecycline	1418	1	2	0.06–16	92.6	6.3	1.1	74.7	17.1	8.2
Amikacin	1418	16	64	0.25–64	52.1	28.1	19.8	30.3	13.2	56.6
Colistin	1046	1	16	0.06–16	NA	NA	NA	74.2	0	25.8
Aztreonam	1418	256	256	0.03–256	4.4	0.4	95.1	2.7	0.2	97.1
*CRECL*										
Ceftaroline	149	256	256	0.06–256	4.7	0.7	94.6	1.6	0	98.4
Ceftazidime–avibactam	149	128	256	0.06–256	42.3	0	57.7	21.9	0	78.1
Ceftazidime	149	256	256	0.12–256	8.7	1.3	89.9	1.6	1.6	96.9
Cefepime	149	32	32	0.12–32	14.8	10.7	74.5	4.7	0	95.3
Pip-taz	149	256	256	2–256	10.1	5.4	84.6	1.6	1.6	96.9
Doripenem	149	8	16	0.06–16	14.1	6.7	79.2	0	0	100
Imipenem	149	8	16	4–16	0	0	100	0	0	100
Meropenem	149	8	16	0.03–16	14.1	12.1	73.8	1.6	17.2	81.3
Levofloxacin	149	4	16	0.03–16	43.6	7.4	49	17.2	4.7	78.1
Tigecycline	149	1	4	0.12–8	83.9	14.1	2	59.4	15.6	25
Amikacin	149	4	64	0.5–64	80.5	5.4	14.1	60.9	7.8	31.3
Colistin	118	0.5	1	0.12–16	NA	NA	NA	93.8	0	6.3
Aztreonam	149	64	256	0.06–256	24.2	2	73.8	18.8	6.3	75
*CRPAE*										
Ceftaroline	4546	128	256	0.015–256	NA	NA	NA	NA	NA	NA
Ceftazidime–avibactam	4546	4	64	0.015–256	74.5	0	25.5	70.6	0	29.4
Ceftazidime	4546	16	128	0.12–256	46.6	8.2	45.3	41.4	0	58.6
Cefepime	4546	16	32	0.25–32	47.2	23.2	29.6	41.5	0	58.6
Pip-taz	4546	64	256	0.25–256	34.7	24.8	40.5	29.4	0	70.7
Doripenem	4545	8	16	0.03–16	15.1	22.8	62.1	1.8	5.9	92.3
Imipenem	4546	16	16	8–16	0	0	100	0	0	100
Meropenem	4546	16	16	0.06–16	11.7	15.4	72.9	4.9	33.5	61.7
Levofloxacin	4546	8	16	0.015–16	36	10	54	22.1	0	77.9
Tigecycline	4546	16	16	0.03–16	NA	NA	NA	NA	NA	NA
Amikacin	4546	8	64	0.25–64	72.8	7.3	19.9	60.5	9.2	30.4
Colistin	3521	1	2	0.06–16	96.4	0	3.6	96.6	0	3.4
Aztreonam	4546	16	128	0.06–256	30.7	21	48.3	1.1	46.6	52.4
*CRABA*										
Ceftaroline	2318	256	256	2–256	NA	NA	NA	NA	NA	NA
Ceftazidime–avibactam	2318	64	256	0.06–256	NA	NA	NA	NA	NA	NA
Ceftazidime	2318	128	256	1–256	5.1	2.3	92.7	NA	NA	NA
Cefepime	2318	32	32	0.25–32	3.6	10.7	85.7	NA	NA	NA
Pip-taz	2318	256	256	4–256	0.4	0.7	99	NA	NA	NA
Doripenem	2318	8	16	0.5–16	0.2	0.4	99.4	0	0	100
Imipenem	2318	16	16	8–16	0	0	100	0	0	100
Meropenem	2318	16	16	1–16	0.1	0.5	99.4	0	1.9	98.1
Levofloxacin	2318	8	16	0.06–16	3.8	11.4	84.9	1.4	0.6	98
Tigecycline	2318	1	4	0.03–16	NA	NA	NA	NA	NA	NA
Amikacin	2318	64	64	0.25–64	19.3	7.8	73	16.4	2.7	80.9
Colistin	1552	1	2	0.12–16	92.1	0	7.9	92	0	8
Aztreonam	2318	64	256	2–256	NA	NA	NA	NA	NA	NA
*MRSA*										
Ceftaroline	30100	0.5	2	0.03–64	89.0	10.3	0.7	NA	NA	NA
Ceftazidime–avibactam	30100	64	64	2–64	NA	NA	NA	NA	NA	NA
Ceftazidime	30100	64	64	1–64	NA	NA	NA	NA	NA	NA
Pip-taz	30100	32	32	0.12–32	NA	NA	NA	NA	NA	NA
Doripenem	30100	2	8	0.008–8	NA	NA	NA	NA	NA	NA
Meropenem	30100	4	16	0.015–16	NA	NA	NA	NA	NA	NA
Levofloxacin	30100	4	8	0.015–8	32.4	0.5	67.1	NA	NA	NA
Moxifloxacin	30100	2	4	0.008–8	32.6	3.9	63.5	NA	NA	NA
Minocycline	30100	0.12	8	0.12–16	89.4	5.3	5.4	NA	NA	NA
Tigecycline	30100	0.12	0.5	0.015–4	98.5	1.5	0	NA	NA	NA
Clindamycin	30100	0.12	8	0.03–8	61	0.3	38.7	NA	NA	NA
Erythromycin	30100	8	16	0.12–16	29.7	2.5	67.8	NA	NA	NA
Vancomycin	30100	1	2	0.25–4	100	0	0	NA	NA	NA
Teicoplanin	30100	1	2	0.12–32	100	0	0	NA	NA	NA
Linezolid	30100	2	2	0.5–16	100	0	0	NA	NA	NA
Daptomycin	30100	0.5	1	0.06–4	99.7	0.3	0	NA	NA	NA
Gentamicin	18616	1	64	0.06–64	78.2	0.9	21	NA	NA	NA
Trimethoprim sulfa	18616	0.25	1	0.25–8	95.6	0	4.4	NA	NA	NA
Oxacillin	30100	4	8	4–8	0	0	100	NA	NA	NA
*PRSP*										
Ceftaroline	1925	0.12	0.25	0.008–32	98.2	1.8	0	86.8	0	13.2
Ceftazidime–avibactam	1925	16	64	1–128	NA	NA	NA	NA	NA	NA
Ceftazidime	1925	16	64	1–128	NA	NA	NA	NA	NA	NA
Penicillin	1925	4	8	2–16	0	0	100	0	0	100
Doripenem	1925	1	2	0.015–8	89.4	10.6	0	81.1	0	18.9
Meropenem	1925	1	2	0.008–2	3.4	32.3	64.3	100	0	0
Levofloxacin	1925	1	2	0.12–16	95.3	0.5	4.2	94.1	0	6
Moxifloxacin	1925	0.12	0.25	0.03–8	95.7	1.5	2.8	94.6	0	5.4
Minocycline	1925	4	4	0.03–4	28.5	13.2	58.3	18.8	3	78.2
Tigecycline	1925	0.03	0.03	0.008–2	99.8	0.2	0	NA	NA	NA
Clindamycin	1925	2	2	0.008–2	32.6	0.3	67.2	24.3	0	75.7
Erythromycin	1925	2	2	0.008–2	13.9	0.2	86	10.9	0.1	89
Vancomycin	1925	0.5	0.5	0.015–2	99.9	0.1	0	100	0	0
Linezolid	1925	1	1	0.06–2	100	0	0	100	0	0

*CLSI* Clinical Laboratory and Standards Institute, *EUCAST* European Committee on Antimicrobial Susceptibility Testing, *CRECO* Carbapenem-resistant *Escherichia coli*, *CRKPN* Carbapenem-resistant *Klebsiella pneumonia*, *CRECL* Carbapenem-resistant *Enterobacter cloacae*, *CRPAE* Carbapenem-resistant *Pseudomonas aeruginosa*, *CRABA* Carbapenem-resistant *Acinetobacter baumannii*, *MRSA* Methicillin-resistant *Staphylococcus aureus*, *PRSP* Penicillin-resistant *Streptococcus pneumonia*, *NA* not applicable

^a^Cefepime CLSI susceptibility for Enterobacteriaceae adopted the susceptible, susceptible-dose-dependent, and resistant categories

The susceptibilities to the various antibiotics against Gram-negative bacteria (total, regardless of drug resistance) were in general comparable using CLSI 2019 and EUCAST 2019 breakpoints, except for imipenem and tigecycline against *P. mirabilis* (Table 1). Nevertheless, the susceptibilities of many resistant species were lower using the EUCAST 2019 breakpoints compared with the CLSI 2019 breakpoints. For example, the susceptibilities of CR-*E. coli* (72.3% vs. 40.5%) and *CR-E. cloacae* (42.3% vs. 21.9%) to ceftazidime–avibactam, and the susceptibilities of CR-*E. coli*, CR-*K. pneumoniae*, *CR-E. cloacae*, and CR-*P. aeruginosa* to levofloxacin, tigecycline, and amikacin (all with a > 10% difference) were noticeably lower when the EUCAST 2019 breakpoints were applied (Table 3).

### In vitro activities of ceftaroline and ceftazidime–avibactam against Gram-positive bacteria from 2012 to 2016

In the Gram-positive strains, ceftaroline showed more than 90% susceptibility rates of *S. aureus*, *S. pneumoniae*, α-hemolytic *Streptococcus*, and β-hemolytic *Streptococcus* (CLSI 2019). TheMIC_50_/MIC_90_ of ceftaroline for coagulase-negative *Staphylococcus* and *E. faecalis* were 0.25/1 mg/L and 1/16 mg/L, respectively. Ceftaroline demonstrated low activity against *E. faecium* (MIC_50_/MIC_90_, 64/64 mg/L) (Table 2). Ceftazidime–avibactam showed low activity against coagulase-negative *Staphylococcus*, *S. aureus*,* E. faecalis*, and *E. faecium* (MIC_50_/MIC_90_: 16–64/64 mg/L), moderate activity against *S. pneumonia* and α-hemolytic *Streptococcus* (MIC_50_/MIC_90_, 0.25/16 mg/L), and high activity against β-hemolytic *Streptococcus* (MIC_50_/MIC_90_, 0.025/0.5 mg/L). The addition of avibactam to ceftazidime was not associated with improved activities against the tested Gram-positive strains. For all tested *Staphylococcus*, *Streptococcus*, and *Enterococcus*, high susceptibility (> 90%) to linezolid, tigecycline, daptomycin, and vancomycin were observed (excepted for *E. faecium* to vancomycin). High activities (susceptibility, > 90%) of levofloxacin and moxifloxacin were observed for *Streptococcus.*

Regarding the resistant Gram-positive strains, ceftaroline demonstrated high activities against methicillin-resistant *S. aureus* (CLSI 2019susceptibility, 89.0%) and penicillin-resistant *S. pneumoniae* (CLSI 2019susceptibility, 98.2%), whereas ceftazidime–avibactam demonstrated limited activities (MIC_50_/MIC_90_: 16–64/64 mg/L) (Table 3). For comparator agents, potent activity (CLSI 2019 susceptibility, > 95%) against methicillin-resistant *S. aureus* was observed for linezolid, tigecycline, vancomycin, teicoplanin, daptomycin, and trimethoprim sulfa, whereas the susceptibility of penicillin-resistant *S. pneumonia* (CLSI 2019 susceptibility, > 95%) was high to linezolid, tigecycline, vancomycin, levofloxacin, and moxifloxacin (Table 3).

The susceptibilities of Gram-positive bacteria (regardless of drug resistance) were similar between the CLSI 2019 and EUCAST 2019 breakpoints, except for the susceptibility of coagulase-negative *Staphylococcus* to teicoplanin and gentamicin. In terms of resistant strains, noticeably lower susceptibility of penicillin-resistant *S. pneumoniae* to ceftaroline (98.2% vs. 86.8%) and meropenem (3.4% vs. 100%) was observed using ECUAST breakpoints as compared with CLSI 2019 breakpoints.

### Global trend of the susceptibilities of pathogens against ceftaroline and ceftazidime–avibactam from 2012 to 2016

**Fig. 1:**
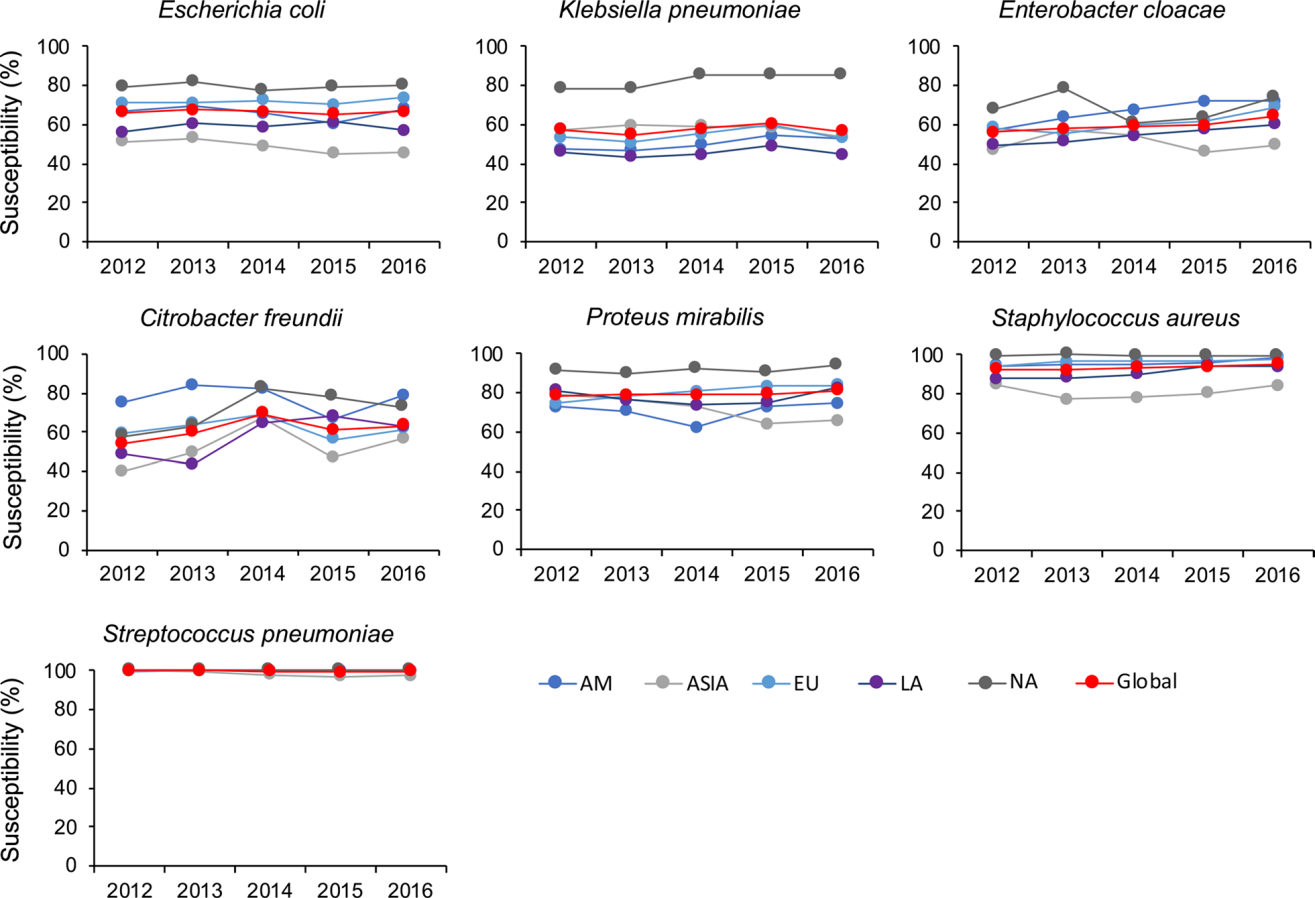
Trends of in vitro susceptibility to ceftaroline against various bacterial species over time in different regions using the CLSI breakpoint. *AM* Africa/Middle-East, *EU* Europe, *LA* Latin America, *NA* North America. Data are not presented for Oceania due to limited number of isolates

Figure 1 presents the trends of susceptibilities to ceftaroline against key bacterial species over time in different regions using the CLSI 2019 breakpoints. For *E. coli* (2012/2016:66.2%/66.5%), *K. pneumoniae* (2012/2016: 57.4%/60.4%), *P. mirabilis *(2012/2016: 78.7%/81.2%), *S. aureus* (2012/2016:92.5%/95.1%) and *S. pneumonia* (2012/2016:99.9%/99.7%), the overall global susceptibility to ceftaroline remained relatively stable in all regions from 2012 to 2016, but some decreases were observed in specific areas of the world. For *E. coli*, the susceptibilities were consistently higher in North America (77.1–82.0%) and lower in Asia (45.1–53.0%). Higher susceptibilities in North America were also observed for *K. pneumoniae* and *P. mirabilis*, and lower susceptibilities in Asia were observed for *S. aureus*. For *E. cloacae*, the global susceptibility gradually increased from 56.2% in 2012 to 64.6% in 2016. For *C. freundii*, the global susceptibility peaked at 69.1% in 2014, decreased slightly in 2015, and rebounded to 63.2% in 2016.

**Fig. 2:**
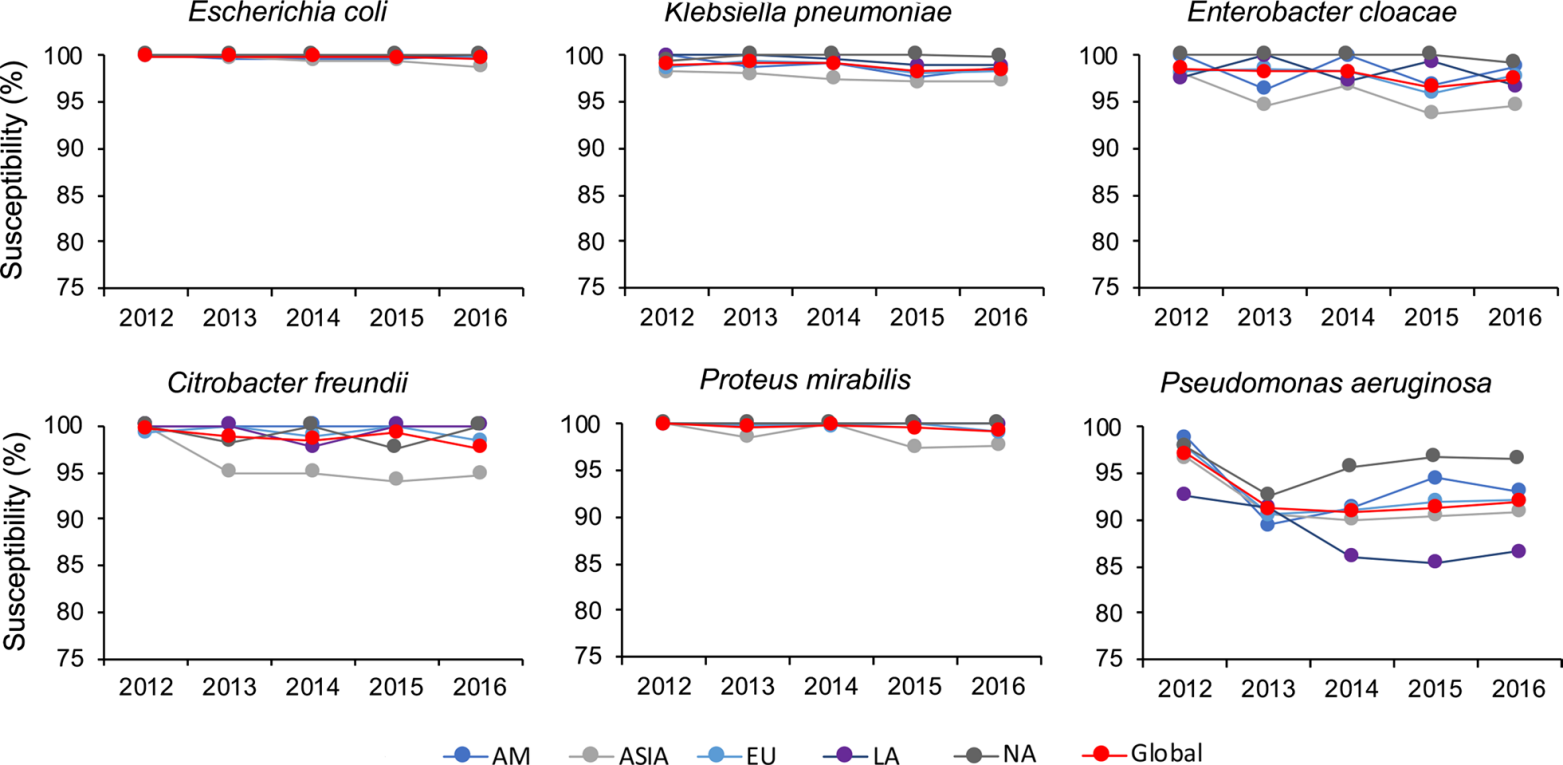
Trends of in vitro susceptibility to ceftazidime–avibactam against various bacterial species over time in different regions using the CLSI breakpoint. *AM* Africa/Middle-East, *EU* Europe, *LA* Latin America, *NA* North America, *OC* Oceania. Data are not presented for Oceania due to limited number of isolates

Figure 2 presents the trends of susceptibility to ceftazidime–avibactam against key bacterial species over time in different regions using the CLSI 2019 breakpoint. The susceptibility of *E. coli*, *K. pneumoniae*, and *P. mirabilis* to ceftazidime–avibactam remained high (> 95%) and relatively stable over time, but with some decreases were observed in specific regions. The susceptibilities of *E. cloacae* and *C. freundii* to ceftazidime–avibactam remained relatively stable over time in all regions, but the susceptibilities in Asia (2013/2016: 94.6%/94.6% and 94.9%/94.7%) decreased in 2013 and were consistently lower than the global rates there after (2013/2016: 98.3%/97.4% and 99.7%/97.6%). The global susceptibilities of *P. aeruginosa* to ceftazidime–avibactam globally decreased from 2012 to 2016 (2012/2016: 97.1%/92.0%), with lower rates observed in Latin America (2012/2016: 92.7%/86.6%), and higher rates observed in North America (2012/2016: 97.9%/96.6%).

### Global trend of the susceptibilities to ceftaroline and ceftazidime–avibactam against multi-drug-resistant species

**Fig. 3:**
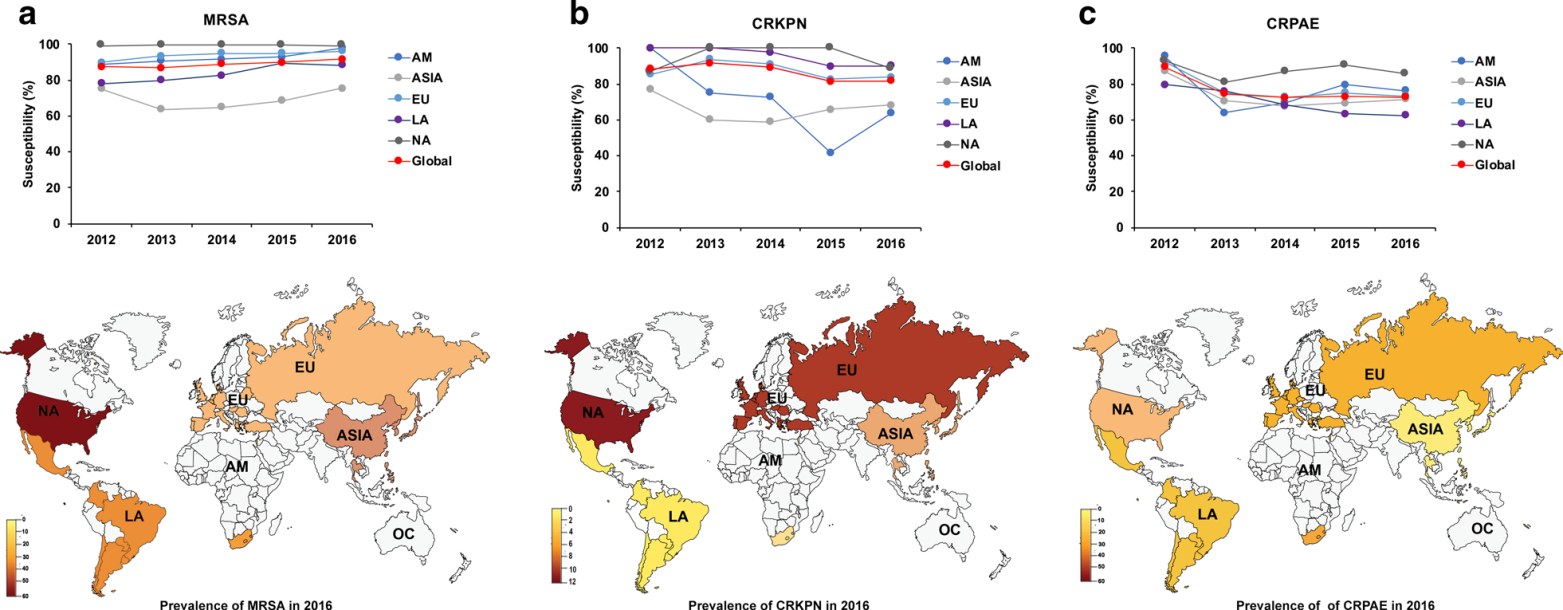
Trends of susceptibility toceftaroline andceftazidime–avibactam against multi-drug resistant bacteria over time in different regions using the CLSI breakpoint. **a** Susceptibility to ceftaroline ofMRSA. **b** Susceptibility to ceftazidime–avibactam of CRKPN. **c** Susceptibility to ceftazidime–avibactam ofCRPAE. *AM* Africa/Middle-East, *EU* Europe, *LA* Latin America, *NA* North America, *MRSA* methicillin-resistant *Staphylococcus aureus*, *CRKPN* carbapenem-resistant *Klebsiella pneumonia*, *CRPAE* carbapenem-resistant *Pseudomonas aeruginosa*. Data are not presented for Oceania due to the limited number of isolates

The proportion of methicillin-resistant *S. aureus* among all *S. aureus* remained stable from 2012 to 2016 (59.8% in 2012 and 2016), with higher prevalence observed in North America (2012/2016: 66.5%/68.1%) and lower prevalence observed in Latin America (2012/2016: 55.9%/53.3%). The overall global susceptibility of methicillin-resistant *S. aureus* to ceftaroline increased slightly from 87.5% in 2012 to 91.7% in 2016, with a marked increase observed in Africa-Middle East (2012/2016: 88.7%/97.8%), Europe (2012/2016: 89.8%/96.2%), and Latin America (2012/2016: 78.2%/88.2%) (Fig. 3a). The susceptibility of methicillin-resistant *S. aureus* to ceftaroline in Asia was consistently lower than in all other regions (2012/2016: 75.2%/75.5%).

The proportion of CR-*K. pneumonia* among all *K. pneumoniae *lightly increased from 6.7% in 2012 to8.2% in 2016, with higher prevalence observed in Latin America (2012/2016: 9.2%/11.2%) and Europe (2012/2016: 9.3%/10.4%). Conversely, the overall global susceptibility of CR-*K. pneumoniae* to ceftazidime–avibactam decreased from 88.4% in 2012 to 81.6% in 2016, with a marked decrease observed in Africa-Middle East (2012/2016: 100%/63.6%), Asia (2012/2016: 76.9%/68.2%), and Latin America (2012/2016: 100%/90%) (Fig. 3b). The susceptibility rates in Asia and Africa-Middle East were, in general, lower than in the other regions during the study period.

The proportion of CR-*P. aeruginosa* among all *P. aeruginosa* remained relatively stable over time (2012/2016: 26.5%/26.7%), with higher prevalence observed for Latin America (2012/2016: 36.3%/34.4%). The overall global susceptibility of CR-*P. aeruginosa* to ceftazidime–avibactam decreased from 89.6% in 2012 to 72.7% in 2016, with a marked decrease observed for all regions (Fig. 3). The susceptibility rate in North America (2012/2016: 93.2%/86.0%) was, in general, higher than in other regions.

## Discussion

Ceftaroline and ceftazidime–avibactam are relatively recent antibiotics that are active against a variety of bacterial species, including some with innate antibiotic resistance [10–13, 15]. The exact resistance patterns to those antibiotics still need to be defined exactly, and there is a crucial need for global surveillance of antibiotic resistance. This study reveals the patterns of the susceptibilities of different bacterial species to a variety of antibiotics, with a focus on ceftaroline and ceftazidime–avibactam, around the world, and over 5 years. The results indicate that the global resistance of CR-*P. aeruginosa* to ceftazidime–avibactam greatly increased over time, while the susceptibility profile of ceftaroline and ceftazidime–avibactam against other species were relatively stable.

The first objective of this study was to examine the overall in vitro activities of ceftaroline and ceftazidime–avibactam using data from the ATLAS program. The results showed that ceftaroline was highly potent (> 90% susceptibility) against Gram-positive strains, including *S. aureus*, *S. pneumoniae*, and *Streptococcus*. On the other hand, ceftazidime–avibactam showed susceptibility > 90% against Gram-negative bacteria, including *Enterobacteriaceae*, *P. aeruginosa*, and *P. mirabilis*, with overtly increased antimicrobial activity observed with the addition of avibactam to ceftazidime. Further analysis of the data from China showed that similar to the global pattern, the susceptibilities of *E. coli*,* K. pneumoniae*, and *P. aeruginosa* to ceftazidime–avibactam were high (92.9–99.0%) in China. Those results are generally similar with those of surveillance studies in China [18], Asia [19], the United States of America [20–22], and Europe [23], and with the AWARE surveillance program [24–26], but with some minute differences that could be due to the specimens’ area of origin since the present study included specimens from all over the world. Another source of difference could be the tested period since bacterial susceptibility changes over time.

Indeed, as shown by the results to the second objective of the present study, the patterns of resistance varied among species, among world regions, and over time. The main differences were that the susceptibility rates of *E. coli* and *S. aureus* to ceftaroline in Asia were lower than the global rates, while those in Europe and North America were generally similar or higher than the global rates. Asia also showed lower susceptibility rates to ceftazidime–avibactam against *C. freundii*, *E. cloacae*, and *P. mirabilis*. A study examined the resistance patterns to ceftaroline, ceftazidime, and piperacillin–tazobactam and revealed similar patterns between Europe and the United States of America [20]. A study across different areas of the United States of America also reported good susceptibility profiles of ceftaroline against respiratory pathogens [27]. A recent report from the World Health Organization revealed high rates of antibiotic resistance all over the world [28, 29]. Antibiotic resistance is a major concern worldwide, and significant differences in the resistance patterns can be observed. The World Health Organization highlighted that even if antibiotic resistance has increased all over the world, the increase was particularly alarming in Asia because of poor health and environment practices such as antibiotic over-prescription, poor infection control, poor waste management, overuse of antibiotics in farming, food security, and restricted access to the newest antibiotics [30-32]. Furthermore, the Asia–Pacific region is the most populous region in the world. Many of its countries are among the poorest, and poor health infrastructure is often encountered [33]. In addition, specific resistance mechanisms (e.g., the New Delhi metallo-β-lactamase-1) are also encountered in Asia [34]. The TEST study showed that Africa and Asia were the two regions of the world with the highest occurrence of *S. aureus* resistant to multiple antibiotics among blood-borne infections [35].

There is a plea for worldwide, automated, and comprehensive surveillance of antimicrobial resistance patterns [8, 36, 37]. Such surveillance could help optimize the worldwide use of antibiotics to improve infection control and minimize the occurrence of resistant strains [38]. In fact, surveillance and proper actions are necessary to avoid medical, social, and economic setbacks that could threaten the very fabric of the global community [38]. Even if the present study focused on ceftaroline and ceftazidime–avibactam, the ATLAS program provides the comprehensive global susceptibility profiles of many antibiotics against a large number of bacterial species. ATLAS receives data from all regions of the world and covers many years. Therefore, it helps provide certain help for the global surveillance of bacterial resistance.

This study has limitations. First, this was a retrospective study, with the inevitable confounding biases, such as the nature of the participating hospitals (mostly tertiary university-affiliated centers), the exact patient populations consulting at those hospitals, and the lack of many variables at the patient level. Second, this study is purely descriptive. Because of the large sample size, minute non-clinically significant differences in susceptibility could be statistically significant, which could be misleading [39, 40]; therefore, statistical tests were not performed.

## Conclusion

In summary, the present study showed that the addition of avibactam improved the activity of ceftazidime against *Enterobacteriaceae* and *P. aeruginosa*. The global antimicrobial susceptibilitiestoceftaroline and ceftazidime–avibactam were, in general, stable from 2012 to 2016, but a marked reduction in the susceptibilities of specific species and CR-*P. aeruginosa* for ceftazidime–avibactam was observed in specific regions of the world.
